# Tc1 mouse model of trisomy-21 dissociates properties of short- and long-term recognition memory

**DOI:** 10.1016/j.nlm.2016.02.002

**Published:** 2016-02-08

**Authors:** Jessica H. Hall, Frances K. Wiseman, Elizabeth M.C. Fisher, Victor L.J. Tybulewicz, John L. Harwood, Mark A. Good

**Affiliations:** a School of Psychology, Cardiff University, CF10 3AT, UK; b Department of Neurodegenerative Disease, UCL Institute of Neurology, Queen Square, London WC1N 3BG, UK; c Francis Crick Institute, The Ridgeway, Mill Hill, London NW7 1AA, UK; d Imperial College, London W12 0NN, UK; e School of Biosciences, Cardiff University, Museum Avenue, Cardiff CF10 3AX, UK

**Keywords:** Tc1, Trisomy-21, Recognition, Spatial, Memory, Perirhinal

## Abstract

The present study examined memory function in Tc1 mice, a transchromosomic model of Down syndrome (DS). Tc1 mice demonstrated an unusual delay-dependent deficit in recognition memory. More specifically, Tc1 mice showed intact immediate (30 sec), impaired short-term (10-min) and intact long-term (24-h) memory for objects. A similar pattern was observed for olfactory stimuli, confirming the generality of the pattern across sensory modalities. The specificity of the behavioural deficits in Tc1 mice was confirmed using APP overexpressing mice that showed the opposite pattern of object memory deficits. In contrast to object memory, Tc1 mice showed no deficit in either immediate or long-term memory for object-in-place information. Similarly, Tc1 mice showed no deficit in short-term memory for object-location information. The latter result indicates that Tc1 mice were able to detect and react to spatial novelty at the same delay interval that was sensitive to an object novelty recognition impairment. These results demonstrate (1) that novelty detection *per se* and (2) the encoding of visuo-spatial information was not disrupted in adult Tc1 mice. The authors conclude that the task specific nature of the shortterm recognition memory deficit suggests that the trisomy of genes on human chromosome 21 in Tc1 mice impacts on (perirhinal) cortical systems supporting short-term object and olfactory recognition memory.

## 1 Introduction

Down syndrome (DS) is an aneuploidy syndrome caused by a trisomy of human chromosome 21 (Hsa21; Chapman & Hesketh, 2000). Approximately 95% of individuals with DS have 47 chromosomes as opposed to 46 that are present in the typical population. The remaining 5% of DS cases are caused by translocation, or partial trisomy (Desai, 1997). DS is the most common genetically defined cause of intellectual disability, with individuals experiencing cognitive impairments, including deficits in learning and memory (Silverman, 2007). Individuals with DS have an increased risk of developing early-onset Alzheimer’s disease (AD), which is thought to reflect, at least in part, overexpression of the amyloid precursor protein (APP; Beyreuther et al., 1993). In order to understand the mechanism(s) by which trisomy of chromosome 21 impacts intellectual development and memory function, various mouse models of trisomy 21 have been developed (Ruparelia, Pearn, & Mobely, 2013). The Tc1 mouse is unique in that it is a transchromosomic line that carries a freely segregating and almost complete copy of human chromosome 21 (Wiseman, Alford, Tybulewicz, & Fisher, 2009). Consistent with the impact of Hsa21 trisomy in humans, Tc1 mice show reduced long-term potentiation (LTP) in the hippocampal dentate gyrus region (O’Doherty et al., 2005) and impaired performance on tasks such as object recognition memory. However, unlike individuals with DS, Tc1 mice are not trisomic for *APP* (Gribble et al., 2013) and thus they provide an opportunity to evaluate the contribution of chromosome 21 genes to cognition in the absence of APP-related brain changes.

Morice et al. (2008) reported that Tc1 mice displayed a deficit in object recognition memory following a delay of 10-min, but not following a 24-h delay; which supported the conclusion that Hsa21 expression impaired short- but not long-term memory. This finding is in broad agreement with evidence from individuals with DS where verbal short-term or working memory processes are impaired, with relative proficiency in visuo-spatial short-term memory tasks (Wang & Bellugi, 1994; but see Yang, Conners, & Merrill, 2014). However, it remains unclear whether the deficit in short-term memory in Tc1 mice extends to a different sensory modality and whether memory for the visuo-spatial attributes of objects is relatively proficient. The latter issue is relevant given evidence that Tc1 mice display aberrant hippocampal short-term, but not long-term, synaptic plasticity, abnormal hippocampal spine morphology, and sub-region changes in the connectivity of the DG-CA3 network that contributes to disruption of place-cell activity (O’Doherty et al., 2005; Witton et al., 2015). It is generally acknowledged that a major contribution of the hippocampus to recognition memory is processing object location and context information (Barker & Warburton, 2011). In contrast, there is relatively little evidence that the hippocampus contributes to short-term object memory (see Hammond, Tull, & Stackman, 2004). The evidence for aberrant hippocampal morphology, plasticity and coding of place information (Witton et al., 2015) would suggest that memory for the spatial organisation of objects will be disrupted in Tc1 mice (c.f., Burke et al., 2011; Lenck-Santini, Rivard, Muller, & Poucet, 2005). Therefore, the aim of this study was twofold: First, we examined immediate, short and long-term recognition memory in Tc1 mice for both visual and olfactory information. Second, we examined memory for object-place information to test the hypothesis that aberrant hippocampal function in Tc1 mice would disrupt memory for the spatial organisation of objects.

## 2 Materials and methods

### 2.1 Subjects

Male Tc1 mice and their age-matched wild type (WT) male litter mates were bred at the Francis Crick Institute, London, transferred to Cardiff University, with appropriate legal documentation, at ~2 months of age and tested at 4–7 months of age. The average weight of the animals was 35 g. Animals were kept on a 12-h light/dark cycle, and all testing was conducted during the light phase of the cycle. Animals were kept in a temperature and humidity controlled environment and were maintained on *ad libitum* access to food and water. Each cage was provided with environmental enrichment in the form of cardboard nesting tubes and wood chew sticks. Tc1 and WT litter mates were housed together in groups of 2–4 per cage. The Tc1 and WT mice used in these experiments were generated from the mating of C57BL/6Jx129S8 (F2) Tc1 females, with C57BL/6Jx129S8 (F1) males. The genotype of the mice was determined by polymerase chain reaction analysis on tissue samples taken from the mice at weaning. (Tc1-specific primers forward: 5′-GGTTTGAGGGAACACAAAGCTTAACTCCCA-3′; reverse: 5′-ACAGAGCTACAGCCTCTGACACTATGAACT-3′; control primers forward: 5′-TTACGTCCATCGTGGACAGCAT-3′; reverse: 5′-TGGGCTGGGTGTTAGTCTTAT-3′).

Three separate cohorts of animals were used in the current study. Experiment 1a was conducted with a cohort of 26 animals (12 WT and 13 Tc1 mice); Experiments 1b, 2a and 2b were conducted on a new cohort of 16 animals (8 WT and 8 Tc1). The interval between experiments was approximately one week. Experiment 3 was conducted on a new cohort of 24 animals (12 WT and 12 Tc1 mice).

Experiment 4, used 11 heterozygous male Tg2576 mice that expressed the “Swedish” amyloid precursor protein mutation (HuAPP_695_SWE; driven by a hamster prion protein promoter; cf. Hsiao et al., 1996) together with 10 WT male litter mate control mice, maintained on a hybrid background of C57BL/6 x SJL. The genotype of the mice was determined by taking ear clips., The tissue was then analysed using polymerase chain reaction (Tg2576 specific primers: 1502: 5′-GTGGATAACCCCTCCCCCAGCCTAGACCA-3′; 1503B: 5′-CTGACCACTCGACCAGGTTCTGGGT-3′; 1501: 5′-AAGCGGCCAAAGCCTGGAGGGTGGAACA-3′). Transgenic and WT mice were tested at the age of 10–11 months, with an average weight of 28 g. This age range was selected because Tg2576 mice display robust memory deficits at this age point (Barnes, Hale, & Good, 2004). All Tg2576 and WT mice were housed individually, with environmental enrichment in the form of card board nesting tubes and wood chew sticks. Mice were housed individually because of male aggression and the need to maximise survival rates. We acknowledge that individual housing, albeit through necessity, may have an impact on the behavioural phenotype of Tg2576 and WT mice. Nevertheless, the cognitive phenotypes we have reported previously and in the present study are similar to other published reports with this mouse line.

All experiments were performed in accordance with the UK Animals (Scientific Procedures) Act (1986) and associated guidelines, as well as European Union directive 2010/63/EU. The programme of work was also approved by the local ethical review committee at Cardiff University, UK.

### 2.2 Apparatus

The apparatus used for all experiments was a large Perspex arena, 60 × 60 × 40 cm, with a pale grey floor and clear walls, which for the purpose of this experiment were covered with white paper. The box was placed on a square table at waist height. The apparatus was set up in a quiet and brightly lit (38 cd/m^2^ at the arena surface) behavioural testing room. Exploration was recorded with an overhead camera. The camera input was used to monitor activity in the arena on a television monitor and each session was recorded using a Philips DVDR recorder.

The duration of object exploration throughout the trials was recorded manually with a stopwatch. All objects used were everyday objects made of non-porous materials. All objects were at least 10 cm high to avoid the mice climbing and sitting on the objects, and were all weighted so that they could not be displaced by the animals. Both the arena and the objects (including novel objects) were cleaned thoroughly with water and ethanol wipes in between each trial in order to prevent the use of odour cues, urine and excrement were also removed from the arena after each trial.

For the olfactory recognition experiment, odour cubes (Dale Air Ltd, UK) were used. Odour cubes were 5 × 5 × 5 cm and red in colour with holes placed in one surface. The scents used were strawberry, coconut, banana, lime, mint, ginger, cinnamon and coriander.

### 2.3 Experimental design

The week prior to testing, mice were handled for 5 min a day. For three days prior to testing, mice were placed in the behavioural test room in their home cages, for 30 min a day. Mice were also given one habituation session in which to freely explore the arena with no objects present for 10 min. Training commenced the following day. In order to provide comparability with Morice et al. (2008), the mice were presented with three objects (or odour cubes) during the sample and test trials. The sample stage comprised two 10-min sample phases, each separated by a 10-min interval (spent in the home cage located in the testing room). In all experiments, mice received a ten-min test phase following a delay interval. The order of presentation of experimental conditions, and the spatial location of objects was counterbalanced amongst mice in order to avoid order effects or spatial biases.

For each experiment, the dependent variable was the amount of time spent by the animals exploring objects. Object exploration was defined as the time spent attending to (actively sniffing or interacting with) the object at a distance no greater than 1 cm. Object exploration was not scored if the animal was in contact with but not facing the object, or if it attempted to climb on the objects to look around the rest of the arena. In order to ensure that procedures were sensitive to differences between the groups independent of variation in individual contact times, a discrimination ratio was calculated for each experimental test phase and these are described in the appropriate methods section. A value close to 1 indicated a strong preference for the target object, whereas a value of 0.5 indicated no systematic bias for the target object.

### 2.4 Behavioural methods

#### 2.4.1 Experiment 1a: Tc1 novel object recognition following a 10-min or 24-h delay

**Fig 1:**
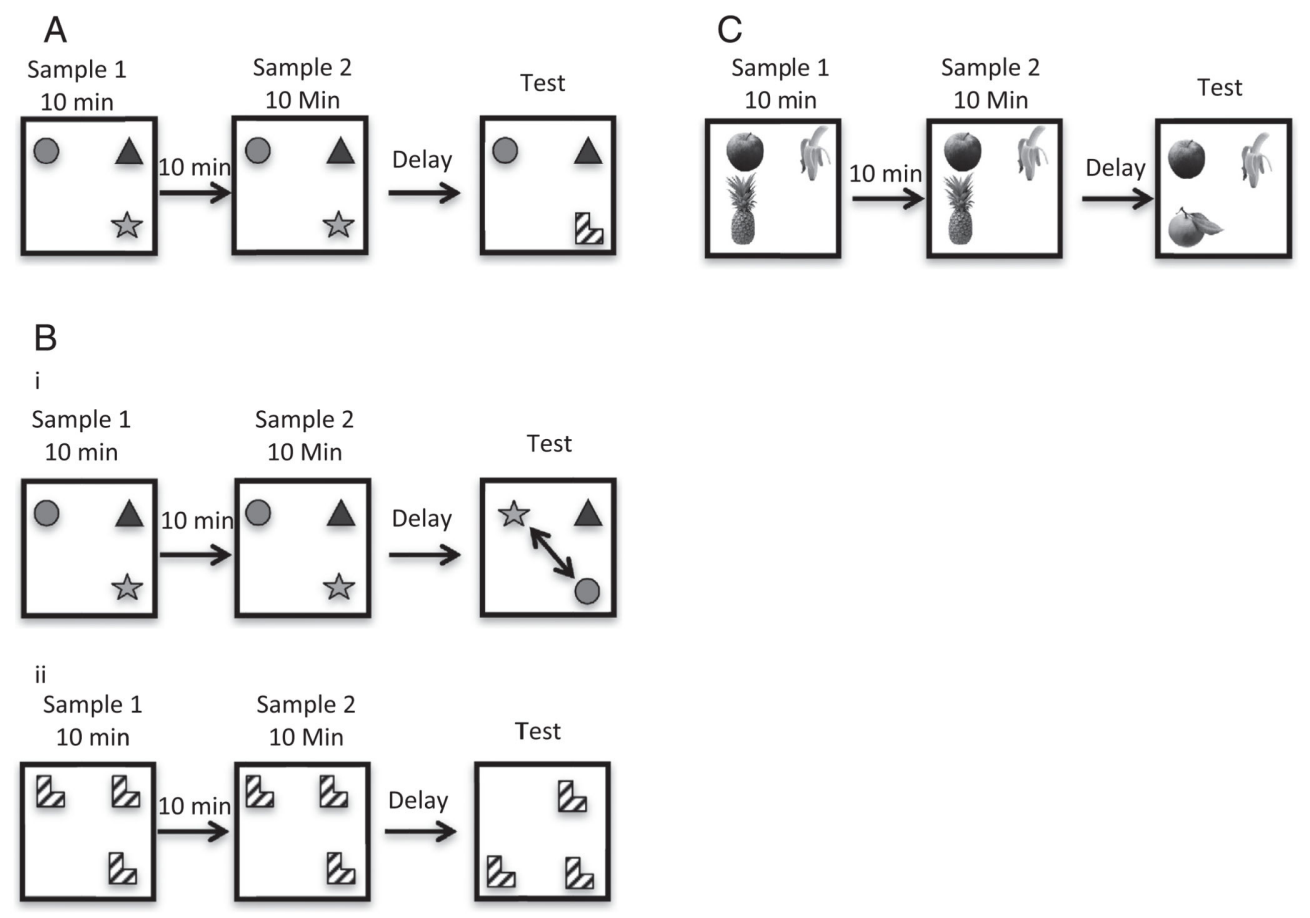
(A) Novel object recognition tasks. Mice were exposed to three objects during two 10-min sample phases. After either an immediate (approximately 30 sec), 10-min, or 24-h delay, mice were returned to the arena for the test phase, during which one of the objects was replaced with a novel object. (Bi) Object-in-place task. Mice were exposed to three objects during two 10-min sample phases. After a delay of either 24-h, or immediately following the sample stage, mice were returned to the arena for the test phase. During the test, two of the objects swapped their spatial location (see arrow). (Bii) Object location task. Mice were exposed to three identical objects during two 10-min sample phases. After a delay of 10-min, mice were returned to the arena for the test phase, during which one of the objects was moved from its original location to a previously vacant corner of the arena. (C) Novel Odour Recognition. Mice were exposed to three visually identical cubes each containing a different odour during two 10-min sample phases. After a delay of either 10-min or 24-h, mice were returned to the arena for the test phase. During the test, one of the odour cubes was replaced with a cube containing a novel odour.

Mice were placed in the centre of the arena and presented with three different objects, each in a different corner of the arena. Mice were allowed to explore the arena and the objects for ten minutes before being removed for a 10-min interval spent in their home cage. Mice were then given a second 10-min sample phase. Following the second sample phase, the mice were returned to their home cage for either a 10-min or 24-h retention interval. In the test phase, one of the items was replaced with a novel object (see Fig. 1a). The time mice spent exploring the novel object, and the time spent exploring the two familiar objects was recorded. The location of the objects and the object that was replaced with the novel item was fully counterbalanced both within and between groups. All objects (novel and familiar) and the arena were wiped down with a 5% alcohol/distilled water wipes between sample phases and prior to returning the mouse to the apparatus for the test stage. The order in which mice received the 10-min or 24-h delay was counterbalanced. Discrimination ratios were calculated as follows: time exploring the novel object/(time spent exploring the novel object + (average time exploring both familiar objects)).

#### 2.4.2 Experiment 1b: Tc1 novel object recognition following a 10-min or immediate delay

To determine whether the deficit in short-term recognition memory was confined to a delay of 10-min, we compared the effects of a very short (“immediate”) delay between the sample and test trial with a 10-min delay interval. The duration of the “immediate” delay interval was the time taken to remove the mouse from the arena after the last sample trial, place it in its home cage, and replace one of the objects with a novel object and clean all objects and arena. The average time was approximately 30 sec. The order in which mice received the 10-min, or immediate delay was fully counterbalanced. Discrimination ratios were calculated in the same manner as Experiment 1a.

#### 2.4.3 Experiment 2a: Tc1 object-in-Place memory following a 24-h or immediate delay

The main aim of this experiment was to assess whether expression of Hsa21 genes in Tc1 mice influenced memory for specific object-location associations. The two sample phases were identical to those used for the object recognition task. However, in the test phase, two of the objects swapped their spatial locations. This resulted in two familiar objects located in different positions, and one familiar object that remained in its original location (see Fig. 1bi). The delay period before administering the test was either immediate or 24 h. The rationale for selecting these intervals was that Tc1 mice would potentially be unable to discriminate objects following a 10-min delay (see results of Experiments 1a and 1b) and this would confound assessment of place recognition in Tc1 mice. The objects that exchanged their spatial locations were counterbalanced, and the location of the objects in the arena was also counterbalanced to avoid spatial biases. The order in which mice received the 24 h or immediate delay was counterbalanced. Discrimination ratios were calculated as follows: average time exploring the two objects in different locations/((time exploring the object in the same location + (average time exploring the two objects in different locations)).

#### 2.4.4 Experiment 2b: Tc1 novel object location memory following a 10-min delay

The main aim of this experiment was to assess whether Tc1 mice were sensitive to a change in the spatial organisation of objects in the arena that was independent of the ability to discriminate between different objects. We used a delay interval of 10-min to determine whether Tc1 mice were able to detect and react appropriately to novelty following this delay interval. The mice were placed in the centre of the arena and presented with three identical objects, each located in a corner of the square arena. Mice were allowed to explore the arena and the objects for 10-min before being removed and placed back in their home cages for a 10-min interval. Mice were then given a second 10-min sample phase before being placed back in their home cages for a 10-min retention interval prior to a test phase. In the test phase, one of the objects was moved from its original location to the previously vacant corner of the arena (see Fig. 1bii); all objects and the arena were cleaned prior to the mouse being returned for the test phase. The time mice spent exploring the object in the novel location, and the time spent exploring the two objects in the same location were recorded. The location of the objects in the arena, and the object that was moved to the vacant corner were fully counterbalanced. Discrimination ratios were calculated as follows: time exploring the object in the novel location/(time exploring the object in the novel location) + (the average time exploring the two familiar object locations).

#### 2.4.5 Experiment 3: Tc1 novel odour recognition

The main aim of this experiment was to test the generality of the recognition memory deficit in Tc1 mice and thus whether the short-term memory impairment extended to olfactory stimuli. The mice received test trials with visually identical plastic cubes each containing a different scent. Mice were placed in the centre of the arena, and presented with three odour cubes, each in a different corner of the arena. The sample procedure was otherwise identical to Experiment 1a. Following a 10-min or 24-h delay after the last sample trial, the mice received a test trial in which one of the odour cubes was replaced with a novel odour cube (see Fig. 1c). The test phase was identical to that described for Experiment 1a. The location of the odour cubes in the arena and the odour that was replaced for the test trial were counterbalanced, other aspects of the procedure were identical to Experiment 1a. A discrimination ratio was calculated as in Experiment 1a.

#### 2.4.6 Experiment 4a: Tg2576 novel object recognition following a 10-min or 24-h delay

To determine whether the pattern of recognition memory changes in Tc1 mice was specific to expression of Hsa21 and not a non-specific consequence of human gene expression, we examined the performance of aged Tg2576 mice on the same behavioural procedure. Tg2576 mice express a human Swedish *APP* mutation linked to early onset Alzheimer’s disease. Tg2576 mice were also of interest because the overexpression of *APP* is absent in Tc1 mice, unlike Down syndrome individuals, and a comparison between these lines would be of theoretical interest. The same novel object recognition protocol described for Experiment 1a was used with one important change. Previous experiments have shown that Tg2576 mice display lower contact times with objects (see Hale & Good, 2005). This was confirmed in the present study and indeed one transgenic mouse was removed from the experiment because it consistently failed to make contact with the object during a preliminary assessment of exploratory activity (data not shown; this mouse was excluded from all subsequent data analysis; *n* = 10 per group). In order to equate exposure times during each sample stage, the exploration times of WT mice were yoked to those shown by Tg2576 mice. This was achieved by pairing WT and Tg2576 mice together for the duration of the experiment. For each pair, the Tg2576 animal was run first on the task and the contact times during each sample stage recorded. The paired WT mouse was then subsequently run, and allowed to accumulate the same contact times with objects as their yoked transgenic mouse. For WT mice, the experimenter stopped each sample exposure once the cumulative total object exploration times matched that of the yoked Tg2576 mouse. Note, there was no attempt to match exploration times with each individual object. The mouse was free to move around the arena. If a WT mouse did not achieve a comparable contact time during the sample phase, the mouse remained in the arena for a maximum of 10 min. During the test phase, WT and Tg2576 mice were given ten minutes to explore the environment freely.

#### 2.4.7 Data analyses

Statistical analyses were conducted using SPSS (version 20.0). A two-way design was used with between subject factor of group and within subject factor of object type. Interactions were analysed using tests of simple main effects. The α-level was set at *p* < 0.05 for all comparisons. To compare discrimination values against chance, one-sample *t*-tests were carried out against a discrimination ratio value of 0.5.

## 3 Results

### 3.1 Experiment 1a: Tc1 Novel object recognition following a 10-min or 24-h delay

The main aim of this experiment was to test the hypothesis that Tc1 mice will show impaired short- but intact long-term object recognition memory. The contact times for each group of mice during the sample phases (collapsed across retention interval conditions) are shown in Table 1 and contact times during the test phase in Table 2, respectively. Inspection of Table 1 suggests that Tc1 mice showed numerically higher contact times with the objects than WT mice. However, the duration of contact decreased in both Tc1 and WT at a similar rate across the sample phases. An Analysis of variance (ANOVA) with sample phase as the within subject’s factor, and genotype as the between subject’s factor revealed a significant main effect of sample phase on object contact time (*F*_(1, 23)_ = 18.264, *p* < 0.001), but no significant main effect of genotype (*F*_(1, 23)_ = 3.767, *p* = 0.065), and no significant interaction between these variables (*F* < 1, *p* = 0.971). This shows that although the Tc1 mice interacted with the objects more than the WT animals, both groups showed a significant decrease in activity (habituation) from sample phase 1 to sample phase 2.

**Fig 2:**
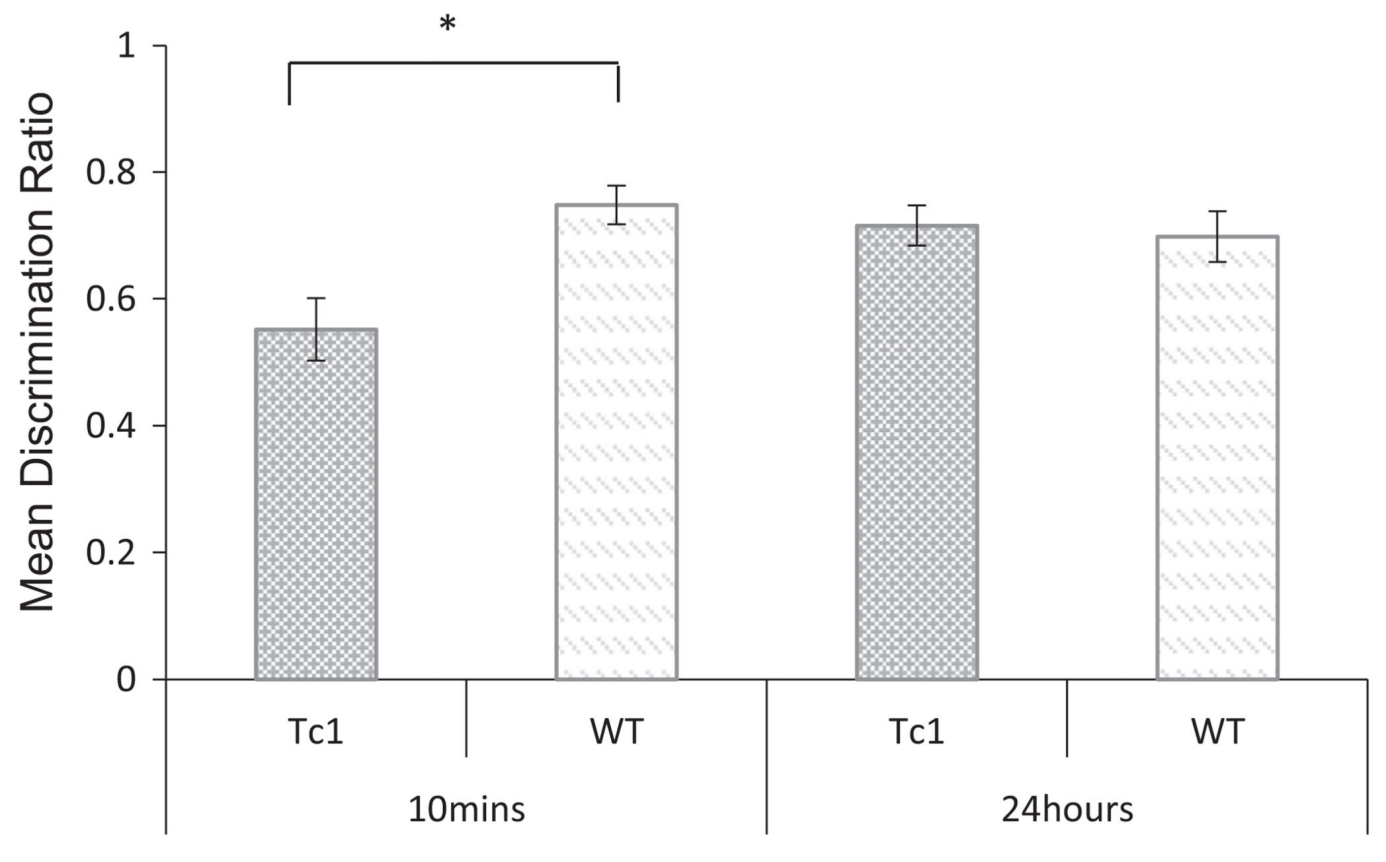
Novel object recognition following a 10-min or 24-h delay in Tc1 and WT control mice. Mean discrimination ratios (error bars represent ± SEM) describing the preference for the novel object for Tc1 and wild type (WT) mice (*p < 0.05).

Table 2 shows the mean contact times with objects (novel and familiar) across the delay conditions for Tc1 and WT mice. A repeated measures ANOVA using object and delay as the within subject’s factors, and genotype as the between subjects factor revealed a significant main effect of object (*F*_(1, 23)_ = 66.156, *p* < 0.001) but no significant main effect of delay (*F* < 1, *p* = 0.567), and no significant main effect of genotype (*F*< 1, *p* = 0.796). The interaction between object and genotype, failed to reach statistical significance (*F*_(1, 23)_ = 3.356, *p* = 0.080). There was no significant interaction between object and delay (*F*_(1, 23)_ = 1.472, *p* = 0.237) or three-way interaction between object, delay and genotype (*F*< 1, *p* = 0.479). In order to evaluate performance that was independent of individual differences in contact times, the data were also analysed using a discrimination ratio and are shown in Fig. 2. Inspection of this figure indicates that wild type control mice discriminated between novel and familiar objects following both a 10-min and 24-h delay. In contrast, the Tc1 mice successfully discriminated between novel and familiar objects only following the 24-h delay. A repeated measures ANOVA using discrimination ratios as the within subject’s factor and genotype as the between subject factor revealed a significant main effect of genotype (*F*_(1, 23)_ = 7.076, *p* < 0.05), but no significant main effect of delay (*F*_(1, 23)_ = 1.726, *p* = 0.202). There was, however, an interaction between these two factors (*F*_(1,23)_ = 6.069, *p* = 0.05). Tests of simple main effects revealed a significant effect of genotype at the 10-min delay (*F*_(1, 23)_ = 11.176, *p* < 0.05), but not at the 24-h delay (*F*< 1, *p* = 0.736). Furthermore, one sample t-test confirmed that the performance of the WT mice were significantly above chance at both delays (10 min: *t*_(11)_ = 8.03, *p* < 0.001; 24 h: *t*_(11)_ = 4.75, *p* < 0.001). However, the performance of Tc1 mice was not above chance at the 10 min delay, (*t* < 1), but was above chance at the 24 h delay (*t*_(12)_ = 6.57, *p* < 0.001). These results therefore confirm that Tc1 mice showed impaired short-term but intact long-term object recognition memory.

### 3.2 Experiment 1b: Tc1 Novel object recognition following a 10-min or immediate delay

The main aim of Experiment 1b was to determine whether Tc1 mice would show a recognition memory deficit when tested immediately after the sample phase. This was to determine whether Tc1 mice had encoded the sample objects as effectively as WT mice. The mean contact times shown by WT and Tc1 mice during the sample phases are shown in Table 1. An ANOVA, with sample phase as the within subject’s factor and genotype as the between subject’s factor revealed a significant main effect of sample phase on contact times (*F*_(1,14)_ = 8.351, *p* < 0.05), but no significant main effect of genotype (*F* < 1, *p* = 0.798), and no significant interaction between these variables (*F* < 1, *p* = 0.431). This result confirmed that both groups interacted with the objects at the same level and habituated to the stimuli across the sample phases to a similar extent.

The mean object contact times during the novelty recognition test are shown in Table 2. Inspection of this table shows that Tc1 mice displayed a normal object novelty preference when tested immediately, but not following a 10-min delay. A repeated measures ANOVA using object and delay as the within subject’s factors, and genotype as the between subjects factor revealed a significant main effect of delay (*F*_(1, 14)_ = 69.037, *p* < 0.001) but no significant main effect of object (*F* < 1, *p* = 0.395) or genotype (*F* <1, *p* = 0.569). There was no significant interaction between object and genotype (*F* < 1, *p* = 0.804). There was, however, a significant three-way interaction between object, delay and genotype (*F*_(1, 14)_ = 5.419, *p* = 0.035). Tests of simple main effects revealed a significant effect of delay on contact times with the familiar object in the Tc1 mice (*F*_(1, 14)_ = 11.569, *p* < 0.005). Tests of simple main effects also revealed a significant effect of object type in WT mice following both the immediate (*F*_(1, 14)_ = 16.075, *p* = <0.01) and 10-min (*F*_(1, 14)_ = 46.144, *p* < 0.001) conditions. There was also a significant effect of object type for Tc1 mice following the immediate test (*F*_(1, 14)_ = 24.380, *p* < 0.001), but not when the test was conducted after 10-min (*F*_(1, 14)_ = 3.912, *p* = 0.068).

**Fig 3:**
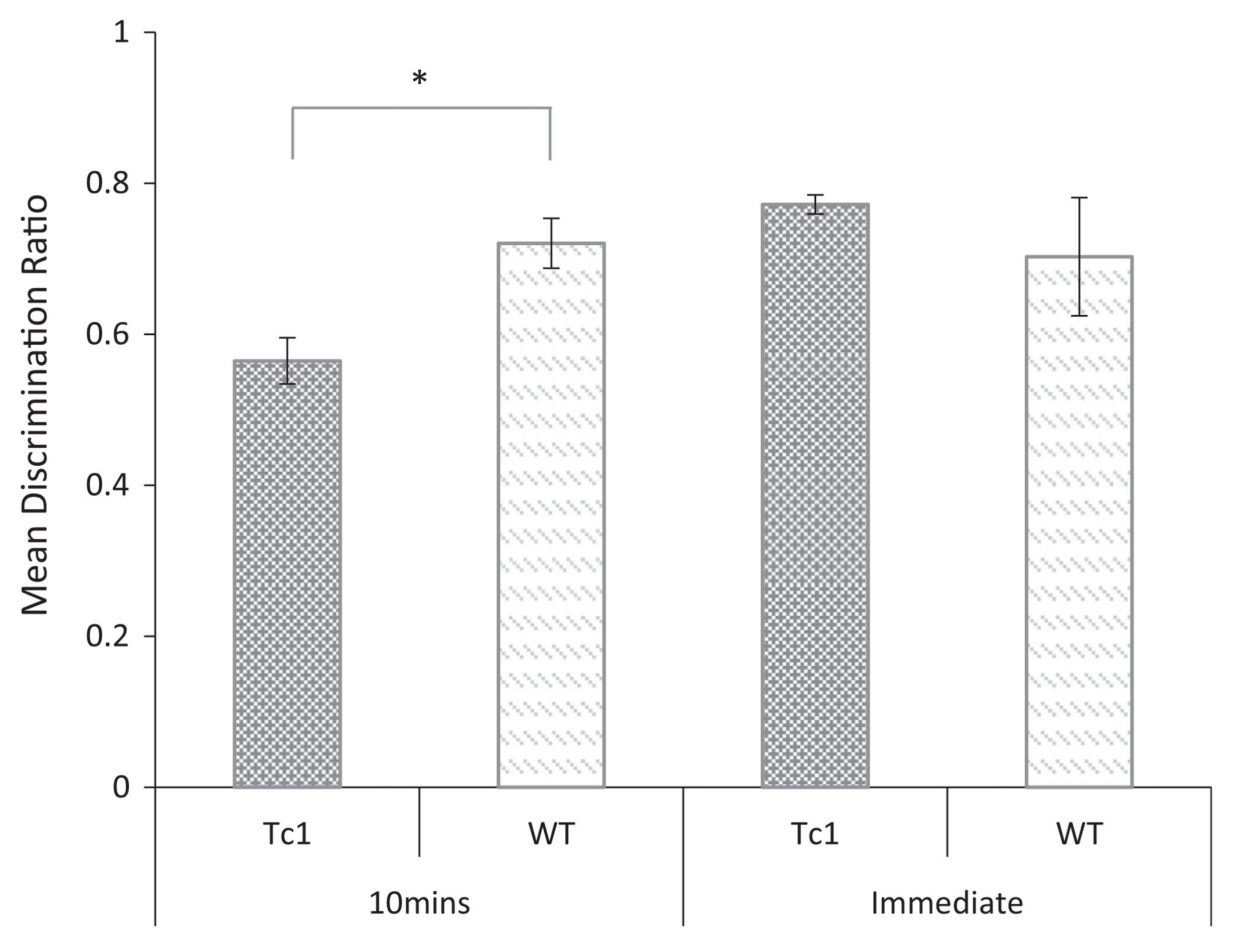
Novel object recognition memory following an immediate or 10 min delay interval in Tc1 and WT mice. Mean discrimination ratios (error bars show ± SEM) for Tc1 and WT mice showing the preference for the novel object following either an immediate or 10-min retention interval (*p < 0.05).

An analysis of the discrimination ratios shown in Fig. 3 revealed a significant main effect of delay (*F*_(1, 14)_ = 5.405, *p* < 0.05), but no significant main effect of genotype (*F* <1, *p* = 0.401). There was however an interaction between these two factors (*F*_(1, 14)_ = 7.642, *p* < 0.05) and tests of simple main effects revealed a significant effect of genotype following the 10-min delay (*F*_(1, 14)_ = 11.986, *p* = 0.004) but not following the immediate delay condition, (*F* < 1, *p* = 0.397). One sample *t*-tests confirmed that the performance of the WT was significantly above chance (0.5) at both delays (10 min: *t*_(7)_ = 6.55, *p* < 0.001; immediate: *t*_(7)_ = 2.52, *p* < 0.05). However, Tc1 mice were above chance in the immediate delay condition only (10 min: *t*_(7)_ = 1.88, *p* = 0.10; immediate: *t*_(7)_ = 19.67, *p* < 0.001). The results of this experiment show that Tc1 mice are able to discriminate novel versus familiar objects following an immediate delay but not when tested after a 10-min interval.

### 3.3 Experiment 2a: Tc1 Object-in-Place memory following a 24-h or immediate delay

The above experiments indicate that Tc1 mice display impaired memory for object information following a short but not long delay interval. The main aim of this experiment was to test the hypothesis that the reported impairment in hippocampal synaptic plasticity and place cell activity in Tc1 mice (O’Doherty et al., 2005; Witton et al., 2015) would disrupt memory for the spatial organisation of objects. The mean contact times with the objects during the sample stages are shown in Table 1. Inspection of these data indicate that WT and Tc1 mice showed a comparable reduction in contact times across sample phases. An ANOVA revealed a significant main effect of sample phase on contact time (*F*_(1, 14)_ = 18.701, *p* < 0.005), but no significant effect of genotype (*F* < 1, *p* = 0.999), and no significant interaction involving these factors, (*F*_(1, 14)_ = 1.105, *p* = 0.311).

Mean contact times with the objects during the test trial are shown in Table 2. WT and Tc1 mice showed comparable exploration of the novel object-location pairings both immediately and 24-h following the last sample trial. A repeated measures ANOVA using object and delay as the within subject’s factors, and genotype as the between subjects factor revealed a significant main effect of object (*F*_(1, 14)_ = 103.726, *p* < 0.001), but no significant main effect of delay (*F*_(1, 14)_ = 2.701, *p* = 0.123) or genotype (*F* < 1, *p* = 0.801).There was a significant delay × genotype interaction (*F*_(1, 14)_ = 4.740, *p* < 0.05). Simple main effects revealed no significant effect of genotype at either the immediate (*F*_(1,14)_ = 1.07, *p* = 0.318) or 24-h condition (*F*_(1, 14)_ = 3.878, *p* = 0.069). The main effect of delay was not significant for Tc1 mice (*F* < 1, *p* = 0.712) but was for WT mice (*F*_(1, 14)_ = 7.298, *p* = 0.05). There was no significant three-way interaction between object, delay and genotype (*F*_(1, 14)_ = 3.39, *p* = 0.087).

**Fig 4:**
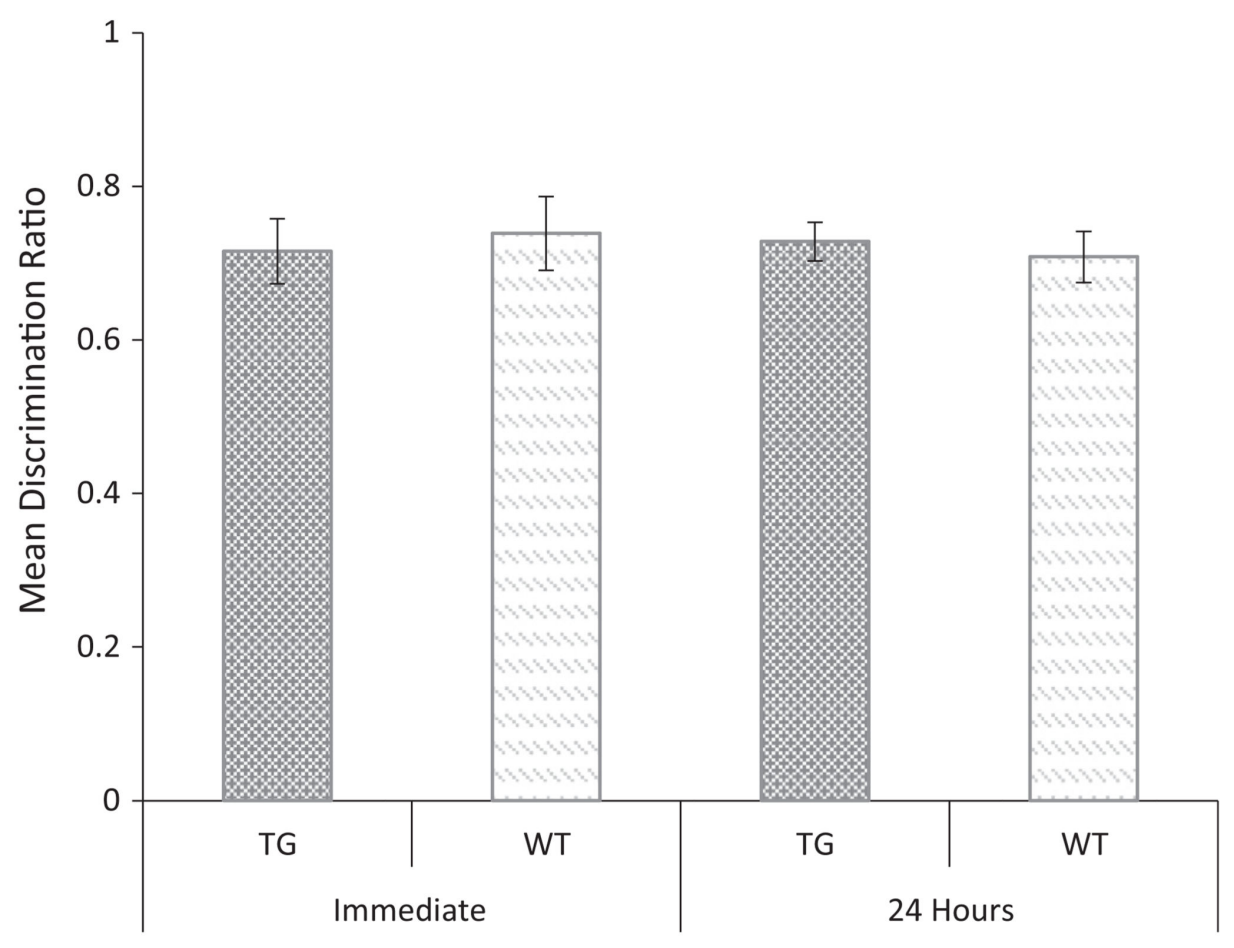
Object-in place memory in Tc1 and WT control mice following an immediate or 24-h delay. Discrimination ratio (error bars represent the ± SEM) describing the preference for the objects in a different (but familiar) location following either an immediate or 24-h delay for Tc1 and WT control mice.

An analysis of the discrimination ratio data (see Fig. 4) demonstrated a similar pattern. An ANOVA showed no main effect of genotype (*F* < 1, *p* = 0.971), or delay (*F* < 1, *p* = 0.735), and no significant genotype × delay interaction (*F* < 1, *p* = 0.408). One sample t-tests confirmed that the performance of both the WT (24 h: *t*_(7)_ = 6.08, *p* < 0.001; immediate: *t*_(7)_ = 4.88, *p* < 0.002) and Tc1 mice (24 h: *t*_(7)_ = 8.77, *p* < 0.001; immediate: *t*_(7)_ = 4.14, *p* < 0.01) were significantly above chance (0.5) at both delays. These results indicate that despite evidence for impaired hippocampal synaptic plasticity and place cell activity in Tc1 mice (Witton et al., 2015), these animals remained sensitive to a mismatch in object-location information following an immediate or 24-h delay.

### 3.4 Experiment 2b: Tc1 Novel object location memory following a 10-min delay

In the previous experiments Tc1 mice showed impaired novelty detection following a 10-min delay. The main aim of the present experiment was to determine whether Tc1 mice were able to react to novelty in a similar manner to WT mice when a familiar object was moved to a completely novel location using the same delay interval. This task does not rely upon the ability to discriminate between objects, as the objects are identical, and is not sensitive to bilateral lesions of the perirhinal cortex but is impaired following hippocampal damage (Barker & Warburton, 2011). The mean contact times for WT and Tc1 mice during the sample stages are shown in Table 1. An ANOVA revealed a significant main effect of sample phase on object exploration (*F*_(1, 14)_ = 9.009, *p* < 0.05), but no significant effect of genotype (*F* < 1, *p* = 0.491), and no significant genotype × sample phase interaction, (*F* < 1, *p* = 0.416).

**Fig 5:**
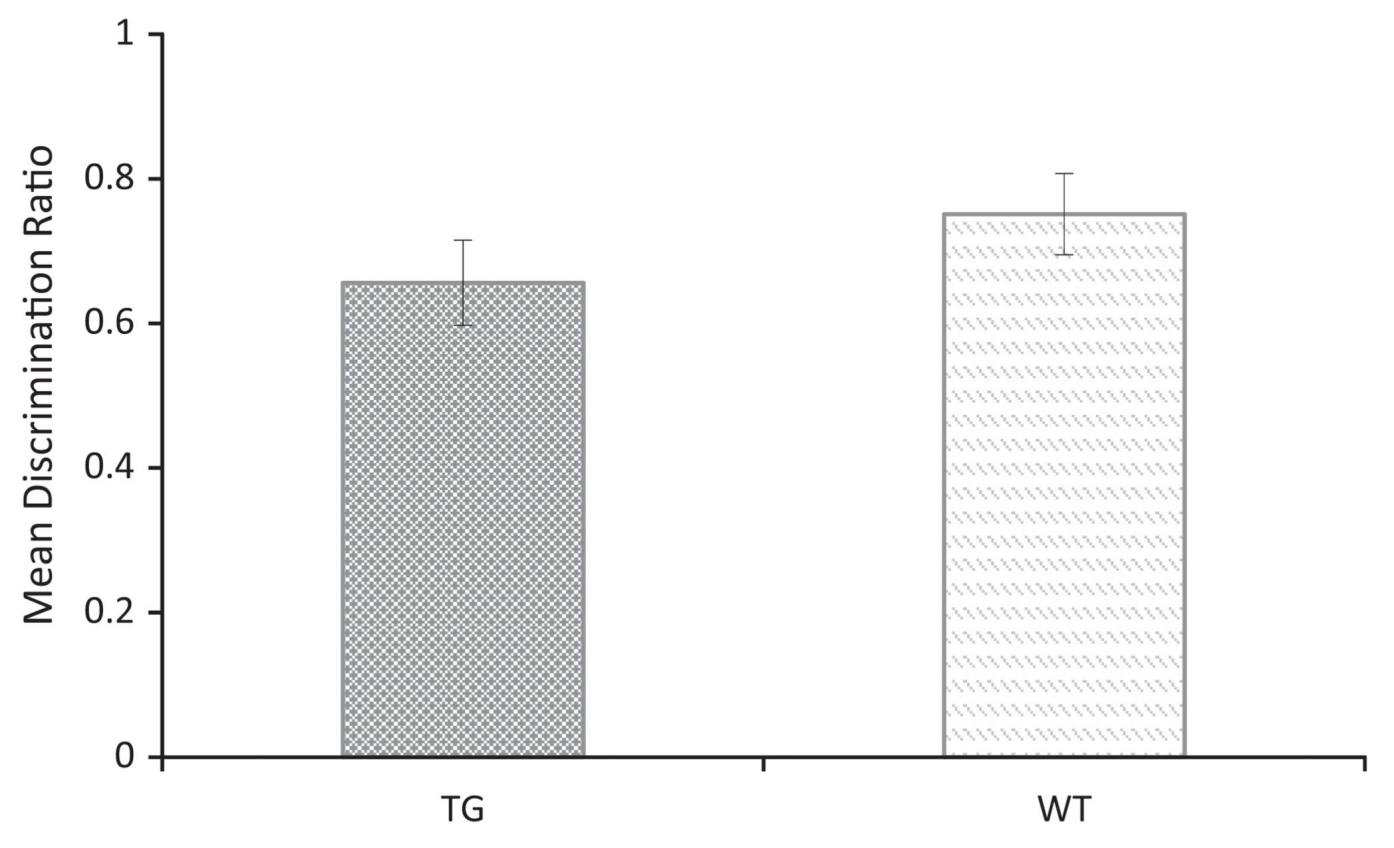
Object location memory following a 10-min delay in Tc1 and WT control mice. Discrimination ratio (error bars ± SEM) describing the preference for the object moved to a completely novel location in the arena.

The mean contact times for both groups during the object location test are shown in Table 2. Inspection of these data show that both WT and Tc1 mice remained sensitive to the movement of a familiar object to a novel location following a 10-min retention interval. An ANOVA revealed a significant main effect of object (*F*_(1, 14)_ = 5.597, *p* = 0.033), but no significant effect of genotype (*F* < 1, *p* = 0.581), and no significant object × genotype interaction (*F*_(1, 14)_ = 3.428, *p* = 0.085). An analysis of the discrimination ratios (see Fig. 5) confirmed the performance of the WT and Tc1 mice was comparable (*t*_(14)_ = 1.169, *p* = 0.262) and that both WT (*t*_(7)_ = 4.4, *p* < 0.01), and Tc1 mice (*t*_(7)_ = 2.55, *p* < 0.05), performed above chance. With reference to experiments 1a, b, the results of the present experiment are important because they show that Tc1 mice were able to process and react to novelty and specifically spatial novelty following a 10-min retention interval. This indicates that the object novelty impairment of Tc1 mice at the 10 min delay is not a refection of a deficit in either detecting novelty or modifying exploratory behaviour following a 10-min retention interval.

### 3.5 Experiment 3: Tc1 Novel odour recognition

The aim of this experiment was to test the hypothesis that the Tc1 impairment in short-term recognition memory was not sensory domain specific. The contact times for WT and Tc1 mice during the odour sample stages are shown in Table 1. An ANOVA revealed a significant main effect of sample phase on contact times (*F*_(1, 22)_ = 15.025, *p* < 0.001), but no significant main effect of genotype (*F* < 1, *p* = 0.790). There was no significant interaction between these variables (*F* < 1, *p* = 0.549). This analysis confirmed that both groups showed contact time habituation across the sample phases.

The mean contact times for the olfactory novelty test are shown in Table 2. Inspection of this table shows that Tc1 mice, unlike WT mice, showed a weaker preference for the novel odour following the 10-min delay relative to the 24 h. delay. An ANOVA revealed a significant main effect of odour (*F*_(1, 22)_ = 86.624, *p* < 0.001), a significant effect of delay (*F*_(1, 22)_ = 7.657, *p* < 0.011), and a significant main effect of genotype (*F*_(1, 22)_ = 4.803, *p* < 0.05). There was also a significant delay × genotype interaction (*F*_(1, 22)_ = 8.473, *p* < 0.008). Tests of simple main effects revealed a significant effect of genotype at the 10-min delay (*F*_(1, 22)_ = 10.492, *p* < 0.004), but not at the 24-h delay (*F* < 1, *p* = 0.728). There was also a significant genotype × odour interaction (*F*_(1, 22)_ = 6.091, *p* < 0.05). Tests of simple main effects revealed a significant effect of genotype on contact with the familiar odour (*F*_(1, 22)_ = 11.606, *p* = 0.003), but not with the novel odour (*F* < 1, *p* = 0.408). There was also a significant three-way interaction between odour, delay and genotype (*F*_(1, 22)_ = 6.979, *p* < 0.05). Tests of simple main effects revealed a significant effect of odour type on the contact times of WT animals, in both the 10-min (*F*_(1, 22)_ = 46.708, *p* < 0.001), and the 24-h delay (*F*_(1, 22)_ = 35.215, *p* < 0.001) conditions. There was also a significant effect of odour on the contact times of Tc1 mice in the 24-h condition (*F*_(1, 22)_ = 31.910, *p* < 0.001), but not in the 10-min delay condition (*F*_(1, 22)_ = 3.292, *p* = 0.083).

**Fig 6:**
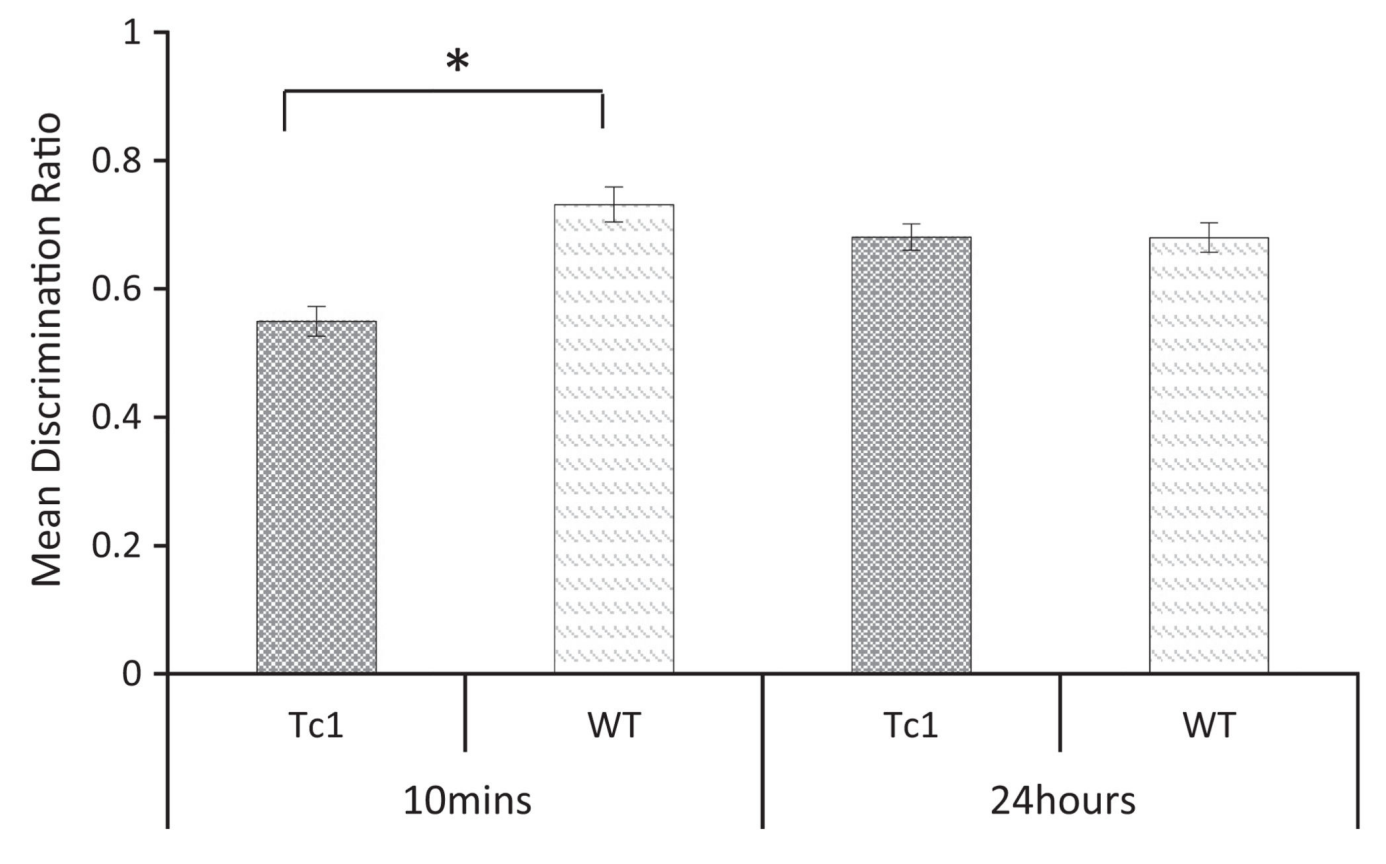
Novel odour recognition memory following a 10-min or 24-h delay in Tc1 and WT control mice. Mean discrimination ratios (error bars represent ± SEM) describing the preference for the novel odour following a 10-min or 24-h delay for Tc1 and WT control mice (*p < 0.05).

An ANOVA carried out on the discrimination ratio data (see Fig. 6) revealed a similar pattern and showed a significant main effect of genotype (*F*_(1, 22)_ = 25.992, *p* < 0.001), but no significant main effect of delay (*F*_(1, 22)_ = 3.103, *p* =0.092) and a significant interaction between these two factors (*F*_(1, 22)_ = 16.228, *p* < 0.01). Tests of simple main effects revealed a significant effect of genotype at the 10-min delay (*F*_(1, 22)_ = 25.992, *p* < 0.001), but not on the 24-h delay (*F*<, *p* = 0.979). One sample *t*-tests confirmed that the performance of the WT mice was above chance at both delays (10 min: *t*_(11)_ 8.29, *p* < 0.001; 24-h: *t*_(11)_ = 7.52, *p* < 0.001). However, the performance of Tc1 mice was above chance only at the 24-h delay (*t*_(11)_ = 8.61, *p* < 0.001) and not following the 10 min delay (*t*_(11)_ = 1.93, *p* = 0.07). These results confirm that Tc1 mice showed impaired short-term but intact long-term recognition memory for odour stimuli.

### 3.6 Experiment 4a: Tg2576 Novel object recognition following a 10-min or 24-h delay

The main aim of Experiment 4a was to determine whether the pattern of impaired short but intact long-term object recognition memory was specific to the Tc1 mouse line or a non-specific effect of the expression of human genes on performance. We therefore examined the effects of the overexpression of a mutant human APP mutation, linked to an early-onset form of Alzheimer’s disease, in Tg2576 mice on object recognition memory. The contact times during the sample phases for WT and Tg2576 mice were yoked, and the mean contact times are shown in Table 1. An ANOVA confirmed that the two groups were matched for contact times during the sample phases, with no significant main effect of genotype (*F* < 1, *p* = 0.672). There was a significant main effect of sample phase on contact times (*F*_(1, 18)_ = 5.636, *p* < 0.05) and no interaction between these factors (*F* < 1, *p* = 0.534). The results confirm that both groups showed a significant decrease in contact times (habituation) across the sample phases.

The mean contact times for both Tg2576 and WT mice during the test phase of the object novelty task are shown in Table 2. A repeated measures ANOVA revealed no significant main effect of delay, (*F* < 1, *p* = 0.565), but a significant main effect of object (*F*_(1, 18)_ = 79.941, *p* = < 0.001), and a significant main effect of genotype, (*F*_(1, 18)_ = 81.300, *p* = < 0.001). There was no significant delay × object interaction (*F* < 1, *p* = 0.454). There was, however, a significant delay × genotype interaction (*F*_(1, 18)_ = 7.493, *p* = < 0.014). Subsequent tests of simple effects revealed a significant effect of genotype at both the 10-min (*F*_(1, 18)_ = 103.274, *p* = < 0.001) and the 24-h delays (*F*_(1, 18)_ = 20.686, *p* = < 0.001); reflecting the overall lower contact times of Tg2576 mice. There was also a significant object × genotype interaction (*F*(_1, 18_) = 33.988, *p* = < 0.001). Simple effects revealed an effect of object for both transgenic (F_(1, 18)_ = 103.274, *p* = < 0.001) and WT mice (*F*_(1, 18)_ = 20.686, *p* = < 0.001). There was a three-way interaction of delay × object × genotype (*F*_(1, 18)_ = 5.093, *p* < 0.05). Simple effects revealed a significant effect of genotype on contact times with the novel object at 10-min (*F*_(1, 18)_ = 19.248, *p* < 0.001), and the familiar object at 10-min (*F*_(1, 18)_ = 16.665, *p* < 0.01). There was also a significant effect of genotype on contact times with the novel object at 24-h (*F*_(1, 18)_ = 60.915, *p* < 0.001) and the familiar object at 24 h (*F*_(1, 18)_ = 15.171, *p* = < 0.001). However, there was no significant effect of delay on contact times with the novel object in Tg2576 mice (*F*_(1, 18)_ = 3.564, *p* = 0.075), or the familiar object (*F* < 1, *p* = 0.830). There was no significant effect of delay on contact times with the novel object in WT mice (*F*_(1, 18)_ = 3.902, *p* = 0.064), but there was a significant effect with the familiar object (*F*_(1, 18)_ = 5.892, *p* < 0.05). There was a significant effect of object type for Tg2576 animals at the 10-min delay (*F*_(1, 18)_ = 11.548, *p* < 0.005), but, importantly, not at the 24-h delay (*F* < 1, *p* = 0.994). There was a significant effect of object type for WT animals following the 10-min delay (*F*_(1, 18)_ = 51.924, *p* < 0.001), and the 24-h delay (*F*_(1, 18)_ = 54.188, *p* < 0.001).

**Fig 7:**
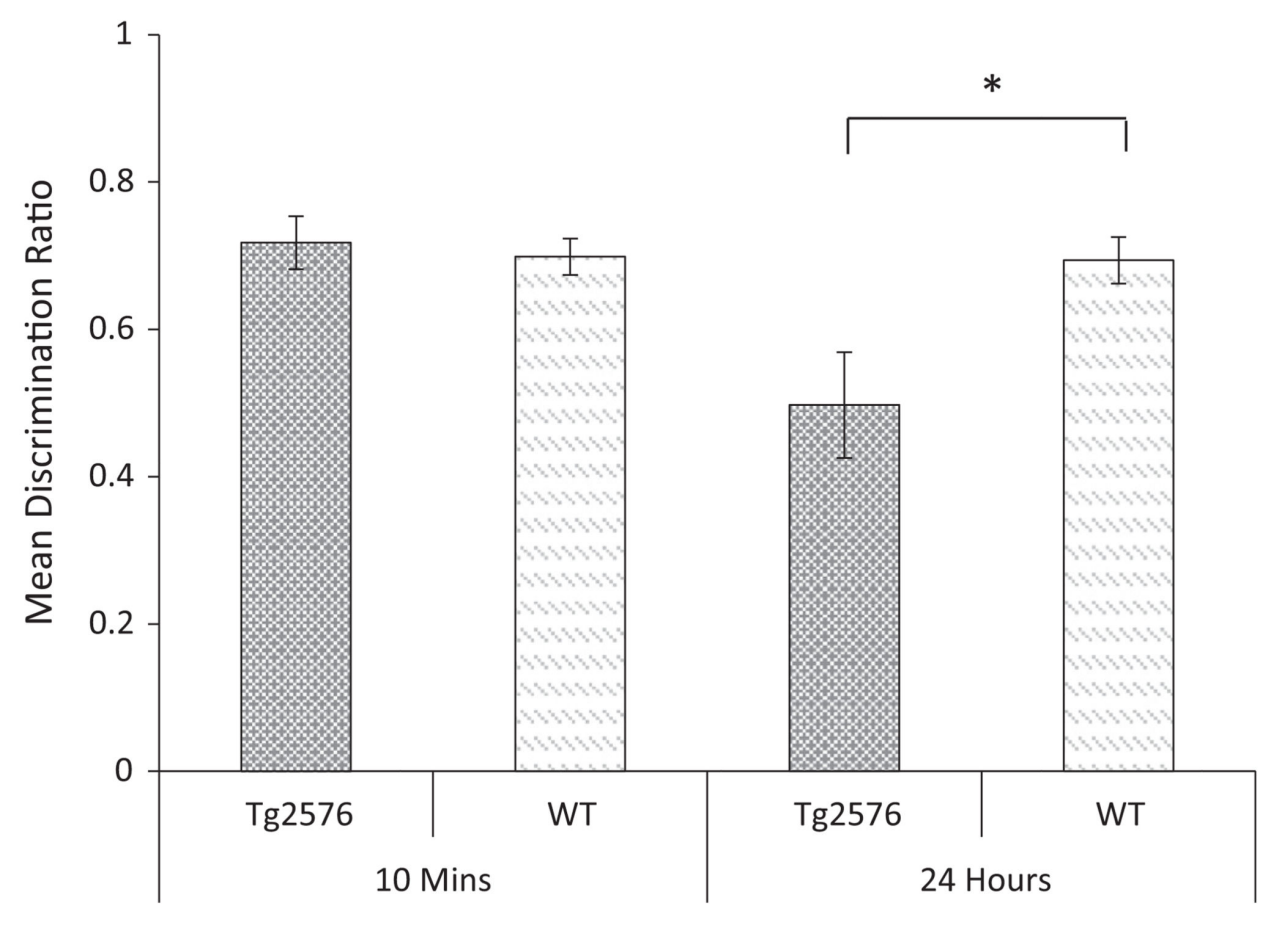
Novel object recognition following a 10-min or 24-h retention interval in Tg2576 and WT control mice. Mean discrimination ratios (error bars represent the ± SEM) describing the preference for the novel object for Tg2576 and WT control mice following either a 10 min or 24-h delay (*p < 0.05).

The discrimination ratio data are shown in Fig. 7. Inspection of this figure shows that Tg2576 mice discriminated between novel and familiar objects following a 10-min delay but not following a 24-h delay. An ANOVA revealed no significant main effect of genotype, (*F*_ (1, 18)_ = 2.863, *p* = 0.108), but a significant main effect of delay, (*F*_(1, 18)_ = 9.903, *p* < 0.01), and a significant genotype × delay interaction, (*F*_(1, 18)_ = 9.060, *p* < 0.01). Tests of simple effects revealed no effect of genotype at the 10-min delay (*F* < 1, *p* = 0.667), but a significant effect of genotype at the 24-h delay (*F*(1, 18) = 6.257, *p* < 0.05). One sample t-tests confirmed that the performance of the WT mice was above chance at both delays (10 min: *t*_(9)_ = 7.66, *p* < 0.001; 24-h: *t*_(9)_ = 5.90, *p* < 0.001). In contrast, the performance of Tg2576 mice was above chance only at the 10 min retention interval (10-min: *t*_(9)_ = 5.93, *p* < 0.001; 24-h: *t* < 1, *p* = 0.93). This pattern was opposite that shown by Tc1 mice and suggests that the recognition deficit in this model was not a non-specific consequence of the expression of human genes.

## 4 Discussion

Tc1 mice express a freely segregating copy of human chromosome 21(e.g., Wiseman et al., 2009). Previous experiments with Tc1 mice have revealed impaired short-term recognition memory, impaired spatial working memory together with impaired LTP induction, altered hippocampal ultrastructure and impaired place cell activity (Morice et al., 2008; O’Doherty et al., 2005; Witton et al., 2015). The present study supported previous findings in demonstrating an impairment in short- but not long-term object recognition memory in Tc1 mice. The current study also established that memory for object novelty was intact when tested immediately after exposure and that the deficit in short-term object recognition memory extended to olfactory stimuli. In contrast to object memory, Tc1 mice showed no impairment in memory for object-place associations (object-in-place task) when tested either immediately or following a 24-h delay. Furthermore, Tc1 mice showed normal place recognition following a 10-min delay using an object location task that minimised the necessity for object discrimination. The latter finding has two implications. First, that the Tc1 deficit in object and odour recognition memory following a 10 min delay was not a result of a general performance deficit or a failure to modify exploratory activity following a 10-min retention interval. Second, while Hsa21 gene expression in Tc1 mice disrupted short-term object memory, it did not impair processing of the spatial attributes of objects at delays of up to 24 h. Finally, the alteration in short-term object memory was specific to Tc1 mice, as Tg2576 mice, that overexpress a human *APP* mutation, displayed intact short-term memory but impaired long-term object memory (see also, Good & Hale, 2007; Oules et al., 2012; Puri, Wang, Vardigan, Kuduk, & Uslaner, 2015).

Before discussing how the expression of human Hsa21 genes in Tc1 mice may have disrupted short-term recognition memory, we first consider the anatomical substrates of recognition memory in normal rodents and its implications for our understanding of memory systems in Tc1 mice. There is considerable evidence that the perirhinal cortex plays a key role in processing object identity (Bussey & Saksida, 2005) and object familiarity/novelty discriminations (Brown & Xiang, 1998). Lesions of the perirhinal cortex impair memory for objects after a delay of 5 min; although recognition memory after a delay of 2 min can remain intact (Norman & Eacott, 2004). Consistent with its key role in processing object identity, lesions of the perirhinal cortex also impairs object-in-place memory, a task that relies on specific object-location associations. In contrast, bilateral lesion of the perirhinal cortex does not impair performance on object-location tasks, where the objects are identical (Barker, Bird, Alexander, & Warburton, 2007; Barker & Warburton, 2011). The contribution of the hippocampus to object novelty remains controversial with some studies reporting deficits in memory at long but not short delays (Broadbent, Squire, & Clark, 2004; Clark, Zola, & Squire, 2000; Hammond et al., 2004) and others reporting no impairment (see Warburton & Brown, 2015, for review). The robust deficit of Tc1 mice in short-term but not long-term object recognition memory suggests that any hippocampal contribute to long-term object recognition memory remained intact. Recent work has illustrated the importance of both the hippocampus and frontal cortex in recognition memory processes. For example, disconnection studies have shown that the hippocampus functions as part of an integrated network with the perirhinal cortex and medial prefrontal cortex supporting object-in-place memory (Barker & Warburton, 2011; Barker et al., 2007) but not object-location memory (Barker & Warburton, 2011; Barker et al., 2007).

A study by Witton et al. (2015) recently reported that Tc1 mice showed abnormal hippocampal place cell activity, hippocampal synaptic morphology and impaired spatial radial-arm working memory. The presence of hippocampal synaptic and place cell deficits suggests that the contribution of this structure to place recognition memory in Tc1 mice should be impaired. The present results clearly contradict this view. Although hippocampal abnormalities in Tc1 mice appear to be sufficient to transiently impair spatial working memory (Witton et al., 2015), they are not sufficient to disrupt processing or memory for the visuo-spatial properties of object arrays, at least using the current testing procedures and parameters. It remains possible, of course, that object-in-place and location memory may be disrupted in Tc1 mice under conditions that place greater demand on memory resources. For example, by increasing the number of object locations or the spatial similarity between object locations (see Smith et al., 2015, for further discussion).

Perhaps one of the most interesting aspects of the present pattern of results is that short-term and long-term recognition processes were dissociated in Tc1 mice. One interpretation of this finding is that in Tc1 mice, cortical systems supporting short-term object memory were disrupted. Consistent with this interpretation, similar dissociations between short- and long-term recognition memory have been reported following manipulation of kainate or cholinergic receptors in the perirhinal cortex. Barker et al. (2006) reported that infusion of a kainate receptor antagonist UBP302 (a selective GLU_K5_ antagonist) into the perirhinal cortex impaired recognition memory following a short (20-min) delay but not following a long (24-h) delay. Antagonism of perirhinal NMDA receptors produced the opposite pattern of results. In other work, Tinsley et al. (2011) showed that antagonism of muscarinic cholinergic receptors in the perirhinal cortex impaired short (20-min), but not long-term (24-h) recognition memory. These results argue for distinct and independent short and long-term memory processes in the perirhinal cortex (Barker et al., 2006). It remains possible that trisomy of Hsa21 genes in Tc1 mice may impact on these cortical receptors. In relation to individuals with DS, there is evidence for polymorphisms in GluK1 kainate receptors (Ghosh, Sinha, Chatterjee, & Nandagopal, 2009). There is also evidence for decreased microtubule motor protein KIF17 expression in trisomic mice, which may alter the distribution of GluK1 localization in distal dendrites (Kayadjanian, Lee, Pina-Crespo, & Heinemann, 2007; Roberson, Toso, Abebe, & Spong, 2008). Although the cholinergic projections to the perirhinal cortex in Tc1 mice have not been characterised, to our knowledge, other DS mouse models, such as Ts65Dn, display age-related changes in the cholinergic system (Ash et al., 2014; Granholm et al., 2002; Kelley et al., 2014). It remains possible that either cholinergic innervation of the perirhinal cortex or expression/activity of perirhinal kainite receptors is altered in Tc1 mice. Clearly further work is required to explore this hypothesis.

The pattern of recognition memory deficits displayed by Tc1 mice differs from that shown by Ts65Dn mice, one of the most commonly used models of DS. Ts65Dn mice are a segmental trisomy model of DS and are trisomic for approximately 56% of genes on mouse chromosome 16 that are homologues for human chromosome 21 (Ruparelia et al., 2013). In contrast to Tc1 mice, several studies have reported Ts65Dn deficits in long-term (24-h) object recognition memory (Colas et al., 2013; De la Torre et al., 2014; Kleschevnikov et al., 2012; Smith, Kesner, & Korenberg, 2014; Stringer, Abeysekera, Dria, Roper, & Goodlett, 2015 Contestabile et al., 2013; Lockrow, Boger, Bimonte-Nelson, & Granholm, 2011; see also Braudeau et al., 2011, who used a 10 min delay). In addition, Smith et al. (2014) reported that Ts65Dn showed impaired memory for object-location information and memory for metric information concerning the distance between objects. Smith et al. (2014) also showed that recognition memory was disrupted in Ts65Dn mice. More specifically, Smith et al. (2014) showed that short-term memory, but not long-term recognition memory, was intact when the Ts65Dn mice were tested in an environment with minimal extramaze cues. When extramaze cues were available, short-term object recognition memory was also impaired in Ts65Dn mice. Other studies have reported object-location memory deficits in Ts65Dn with delays between 10-min-and 24-h (Begenisic et al., 2014; Contestabile et al., 2013; Kleschevnikov et al., 2012; but see Hyde & Crnic, 2002).

The pattern of behavioural deficits in Tc1 mice is therefore clearly different from that shown by Ts65Dn mice. The two models differ in a number of other respects. Tc1 mice express a large part of Hsa21 (approximately 75% of genes; Choong, Tosh, Pulford, & Fisher, 2015); although the mice possess a deletion, 6 duplications and more than 25 de novo structural rearrangements of Hsa21 (Gribble et al., 2013). Ts65Dn possess three copies of the segment of chromosome 16 that is orthologous to a critical region of Hsa21 (Davisson, Schmidt, & Akeson, 1990) and 79 other genes on chromosome 17 that are outside the Hsa21 region of synteny (Choong et al., 2015). One other distinctive difference between the two models is that the amyloid precursor protein (APP) is not trisomic in Tc1 mice, unlike Ts65Dn mice (Choong et al., 2015). This may represent an important difference between these models. APP plays a major role in brain development and neurogenesis and APP trisomy may contribute to abnormal brain development and cognition (Cataldo et al., 2003; Giacomini et al., 2015; Trazzi et al., 2013; see also Choong et al., 2015 for further discussion). In this context, it is is interesting to note that Tg2576 mice showed the opposite pattern of memory deficits to Tc1 mice, indeed a pattern that was arguably more similar to that shown by Ts65Dn mice (see for example Good & Hale, 2007). Although speculative, this pattern of results suggests that aberrant APP expression (perhaps in combination with other genes, see Cataldo et al., 2003) may contribute to impaired long-term object and place recognition memory deficits in Ts65Dn and that other genes may have an impact on short-term (recognition) memory processes. Further behavioural assessment of mouse models trisomic for different regions orthologous to Hsa21 will help to address this question.

The alteration in short-term memory in Tc1 mice is broadly consistent with some changes in memory that are observed in individuals with DS. Impairments in verbal and non-verbal object memory are commonly reported in DS (Vicari, Bellucci, & Carlesimo, 2005, 2006; Vicari, Carlesimo, & Caltagirone, 1995). Furthermore, processing of visuo-spatial information is relatively spared in DS (c.f., Yang et al., 2014); although recent evidence indicates impaired allocentric memory in DS children, consistent with disruption of the hippocampus and related cortical regions (Lavenex et al., 2015; see also Courbois et al., 2013).

In summary, the present study has shown that Tc1 mice possess a selective deficit in short-term recognition memory; a pattern opposite to that shown by Tg2576 mice. These results are consistent with the hypothesis that dissociable neural processes underpin short-term and long-term object recognition memory (Barker et al., 2006). In contrast, both short- and long-term place recognition memory was spared in Tc1 mice. We conclude that the selective disruption of short-term object recognition memory in Tc1 mice points towards aberrant function of cortical systems supporting object memory and may specifically involve the perirhinal cortex. Further studies focusing on cortical changes in Tc1 mice and other segmental trisomy mouse models will help elucidate the mechanisms by which trisomy of genes on human chromosome 21 disrupt memory processes.

## Significance

The importance of this work lies in its demonstration that a mouse model of human trisomy 21 (Down syndrome) shows a selective deficit in short-term recognition memory while sparing long-term memory for the same type of information. Furthermore, the findings are original in showing that the deficit in recognition memory generalises across stimulus modalities (both visual and olfactory) and the pattern contrasts with that shown by a different mouse model overexpressing a human APP mutation linked to familial Alzheimer’s disease. We also show for the first time that the expression of a near complete copy of human chromosome 21 in Tc1 mice does not impair place recognition when using object-in-place or object-location tasks. In conclusion, this work reveals a selective deficit in short-term object and olfactory recognition memory in a mouse model of Down syndrome. The authors conclude that the pattern of behavioural changes suggests that trisomy of genes on human chromosome 21 in mice may cause abnormalities in cortical (perirhinal) systems supporting recognition memory.

## Figures and Tables

**Table 1:** Mean contact times (seconds) during the sample phase with novel and familiar objects for Tc1, Tg2576 and WT control mice.

Genotype	Mean contact time during the sample phases (seconds)	
	Experiment 1a: *Novel object recognition – 10 min vs 24 h delay*	
	
	Sample Phase 1	Sample Phase 2
Tc1	18.12	13.70
WT	13.28	8.93
	Experiment 1b: *Novel object recognition – 10 min vs immediate delay*	
	
	Sample Phase 1	Sample Phase 2
Tc1	30.15	23.12
WT	31.33	20.49
	Experiment 2a: *Object in Place - 24 h versus immediate delay*	
	
	Sample Phase 1	Sample Phase 2
Tc1	23.28	17.31
WT	25.20	15.40
	Experiment 2b: *Object location – 10 min*	
	
	Sample Phase 1	Sample Phase 2
Tc1	26.32	21.32
WT	31.03	22.15
	Experiment 3: *Novel odour recognition – 10 min vs 24 h delay*	
	
	Sample Phase 1	Sample Phase 2
Tc1	13.23	11.03
WT	13.35	10.34
	Experiment 4: *Yoked novel object recognition – 10 min vs 24 h delay in Tg2576 mice*	
	
	Sample Phase 1	Sample Phase 2
Tg2576 & WT	12.44	8.87

**Table 2:** Mean contact times (seconds) during the test phase with novel and familiar objects for Tc1, Tg2576 and WT control mice.

Genotype	Mean contact times during the test stage (seconds)			
	Experiment 1a: *Novel object recognition – 10 min vs 24 h delay*			
	
	Novel object (10 min)	Novel object (24 h)	Familiar object (10 min)	Familiar object (24 h)
Tc1	5.692	7.132	4.048	2.956
WT	6.399	7.716	2.120	2.791
	Experiment 1b: *Novel object recognition – 10 min vs immediate delay*			
	
	Novel object (10 min)	Novel object (Immediate)	Familiar object (10 min)	Familiar object (Immediate)
Tc1	10.910	16.863	8.647	4.083
WT	12.963	11.790	5.190	3.724
	Experiment 2a: *Object in place – 24 h versus immediate delay*			
	
	Novel place (24 h)	Novel place (Immediate)	Familiar place (24 h)	Familiar place (Immediate)
Tc1	9.323	9.909	3.369	3.959
WT	12.422	7.191	5.378	3.215
	Experiment 2b: *Object location – 10 min*			
	
	Novel location (10 min)		Familiar location (10 min)	
Tc1	13.060		5.894	
WT	19.729		4.616	
	Experiment 3: *Novel odour recognition – 10 min vs 24 h delay*			
	
	Novel odour (10 min)	Novel odour (24 h)	Familiar odour (10 min)	Familiar odour (24 h)
Tc1	8.819	7.413	7.580	3.640
WT	7.493	7.209	2.821	3.240
	Experiment 4: *Yoked novel object recognition*			
	
	Novel object (10 min)	Novel object (24 h)	Familiar object (10 min)	Familiar object (24 h)
Tg2576	4.774	1.759	1.924	1.767
WT	10.543	13.698	4.499	6.244
